# Towards a standardized approach of assessing social context of persons receiving home care in Flanders, Belgium: the development and test of a social supplement to the interRAI instruments

**DOI:** 10.1186/s12913-021-06453-w

**Published:** 2021-05-22

**Authors:** Shauni Van Doren, Kirsten Hermans, Anja Declercq

**Affiliations:** 1 LUCAS – Center for Care Research and Consultancy, KU Leuven, Leuven, Belgium; 2 CeSO – Center for Sociological Research, KU Leuven, Leuven, Belgium

**Keywords:** Instrument development, Social context, Social environment, Home care services, Needs assessment, BelRAI Social Supplement, Belgium, interRAI

## Abstract

**Background:**

Apart from a person’s physical functioning, the early identification of social context indicators which affect patient outcomes - such as environmental and psychosocial issues - is key for high quality and comprehensive care at home. During a home care assessment, a person’s biomedical and functional problems are typically considered. Harder to define concepts, such as psychosocial well-being or living arrangements, are not routinely documented, even though research shows they also affect functioning and health outcomes. The purpose of this study is to develop and test a concise, integrated assessment (BelRAI Social Supplement) that evaluates these social context indicators for persons receiving home care to complement existing interRAI- instruments.

**Methods:**

The development of the BelRAI Social Supplement is a multi-stage process, based upon the revised MRC-framework, involving both qualitative and quantitative research with stakeholders such as; clients, informal caregivers, care professionals and policy makers. The developmental process encompasses four stages: (I) item generation based on multiple methods and content validation by a panel of stakeholders (II) assessing feasibility and piloting methods, (III) early evaluation, and (IV) final evaluation. Stage II and III are covered in this paper.

**Results:**

During Stages I and II, a testable version of the BelRAI Social Supplement was developed in an iterative process. In Stage III, 100 care professionals assessed 743 individuals receiving home care in Flanders between December 2018 and December 2019. Using inter-item correlation matrixes, frequency distributions and regular feedback from the participants, the BelRAI Social Supplement was improved and prepared for Stage IV. The updated version of the instrument consists of four main sections: (1) environmental assessment; (2) civic engagement; (3) psychosocial well-being; and (4) informal care and support. In total, the BelRAI Social Supplement contains a maximum of 76 items.

**Conclusions:**

The BelRAI Social Supplement was reviewed and shortened in close collaboration with care professionals and other experts in Flanders. This study resulted in an instrument that documents need-to-know social context determinants of home dwelling adults.

## Background

Over the past years, we observe a continuous rise in the demand for ambulatory care, specifically care at home [1–4]. The rise in the number of older persons with care needs in our communities, and the increasingly complex concurrence of comorbidities creates a pressing need for an update to the health care systems [5, 6]. A more personalized, holistic, multi-disciplinary and outpatient approach to caregiving should replace the standardized, limited, mono-disciplinary and residential tactic [7–11]. Multiple governments adopted the World Health Organization’s (WHO) framework on integrated people-centered health services [12–15]. At the center of this framework, the person with disabilities is surrounded by their family and close community. The person-centered approach values the person and their community as active participants in health and social services, and recognizes the relationship between the individual and other related context [16, 17].

This biopsychosocial model of disability looks beyond the quantitative (medical) diagnosis and impairments, and proposes that a combination of biological, psychological and environmental or social factors influence a person’s functioning and health outcomes [18, 19]. Research shows that social environment determinants such as housing conditions [20], civic engagement [21], socio-economic status [22], and caregiving duties [23] do not always directly impact the (experienced) health and/or disability of a person, but do continuously shape the conditions of daily life [24–26].

One way in which health services can empower persons with disabilities and engage their social network is by defragmenting health systems and facilitating collaboration with organizations and care providers across care settings. This integrated approach to caregiving requires two-way and direct communication between and across all actors in the care landscape [12, 13]. However, the abovementioned shift is putting a lot of pressure on care providers, as their current information systems and structures are not adapted to this way of working [27, 28].

A heterogeneous collection of instruments fosters a fragmented and non-standardized approach to care, as each instrument focusses on a certain issue, problem or care need. A lack of standardized assessment practices hinders an efficient and uniform transition towards an integrated care approach [29–31]. In Belgium, a variety of different instruments and information systems are currently used for assessing a person’s care needs [32, 33]. These instruments evaluate the level of disability or care dependency in various ways, each system using different jargon and highlighting divergent aspects of a person’s functioning based on their primary target population in the care providing organization [27, 34].

Following state reforms, the Belgian regions and communities started upgrading and integrating their (primary) care services, aiming to enhance the effectiveness and efficiency, as well as improve the quality of life of their care users and providers [35]. In 2008, the Belgian government opted for a systematic and mandated implementation of the interRAI instruments in home care and residential care in order to facilitate the process of adopting a uniform, integrated and person-centered care approach and to allow for international comparison [14, 36]. The Belgian communities (Flemish, French and German-speaking Community) are responsible for the actual implementation of the interRAI-instruments^1^.

The interRAI instruments are a collection of internationally validated and comprehensive assessment tools to effectively evaluate persons of different ages with different strengths, preferences and needs. They can be used by a variety of health and social services professionals in different settings (home care, long-term care facility, acute care, etc.) and target groups. The key applications of these instruments include outcome measurement, care planning, quality monitoring and improvement, and resource allocation [37, 38]. Their strength lies within the shared, common language and set of core items. A clinical concept is measured in the same way across the different instruments and - although the instruments are developed with a specific population in mind - they all share a large set of core items. Both the use of common language and an overlap of items allows for the multidisciplinary assessments and efficient transfer of information [39].

The interRAI instruments were translated into Dutch, French and German – the three national languages of Belgium – and were named BelRAI [40]. Home care in Belgium is very accessible, and a large percentage of clients have few problems and do not need complex care. [41, 42]. Caregivers consider a full interRAI Home Care assessment for them too time-consuming with regard to the limited number of areas in which they have problems. A solution to this problem was developing a “BelRAI Screener” in collaboration with the stakeholders. This short-form assessment entirely made up of internationally validated interRAI items focuses on biomedical aspects of functioning and problems with activities of daily living [34]. The BelRAI Screener will be fully implemented in the Flemish home care setting by June 2021 [43].

During the development and piloting of the BelRAI Screener, social care service organizations in Flanders were outspoken advocates for also assessing key contextual factors in addition to the items already in the instrument concerning a person’s physical and mental capabilities and limitations. The social or contextual elements are considered to be equally important for developing a personalized and effective care plan as they play a large role in a person’s experience of disability [34, 44, 45].

This request from care providers to add a ‘social supplement’ to the BelRAI Screener illustrates the shift in health and social care services towards a biopsychosocial model of disability, that emphasizes the interplay of biological, psychological and social factors in a person’s life [46]. The aim of this study is to develop a reliable and valid supplement to the existing BelRAI-instruments in collaboration with relevant stakeholders, in order to assess need-to-know social context themes for adults receiving home care services in Flanders, Belgium.

## Methods

### Study design

The BelRAI Social Supplement is developed and tested using qualitative and quantitative methods. The research design is based on the revised Medical Research Council (MRC) framework for the design, evaluation and implementation of complex interventions [47]. This study is part of a larger evidence-based policy research project to develop a Social Supplement to the existing interRAI instruments “to assess the social context of home-dwelling adults with care needs” [48].

**Fig. 1:**
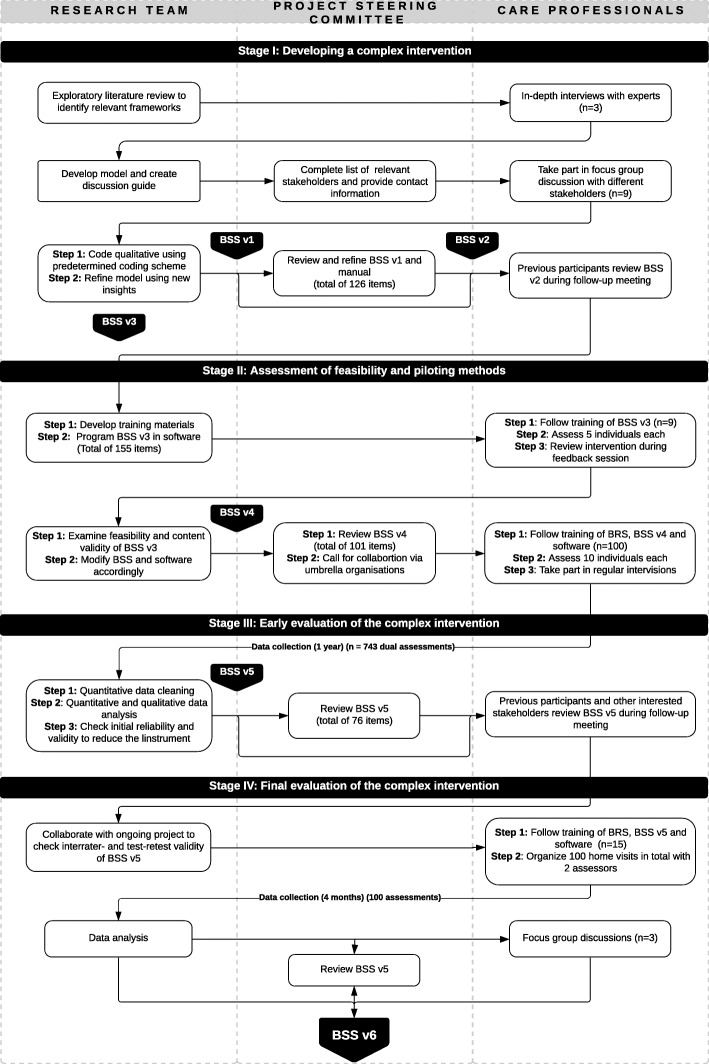
Development and evaluation of the BelRAI Social Supplement based on revised MRC-framework

The research is conducted in four stages during the period of 2017–2020 in close collaboration with care providers. A project steering committee that consisted of experts on care and welfare settings discussed the results at regular intervals during the different stages (Fig. 1). This study covers the results from Stage II (assessment of feasibility and piloting methods) and Stage III (early evaluation via testing of the instrument through a pilot study) of the MRC Framework. Figure 1 offers a more in-depth overview of the different stages and phases in the development and evaluation process.

### Instruments

#### BelRAI Social Supplement (BSS)

 The BelRAI Social Supplement is the assessment we tested and evaluated in this study to assess social context indicators such as the availability of informal caregivers, safe and clean housing, and psychosocial well-being. The test version was developed at the request of, and in cooperation with the Flemish government and the care organizations providing social care services during Stage I of this research. A detailed paper regarding the development of the first three versions of the BelRAI Social Supplement is under review (Van Doren, Hermans and Declercq, unpublished observations). The development of the instrument and training materials was guided by the design principles for the original interRAI instruments: (1) assessments should use all sources of information available; (2) findings should be based on observable traits; (3) concepts and items should have operational definitions and coding instructions that specify inclusion and exclusion criteria; and (4) items should have clearly delimited observation time frames anchored to a specific assessment reference date [37].

In this study, two versions of the BelRAI Social Supplement were used. The version of the BelRAI Social Supplement consisting of 155 items divided into 4 main themes; (a) environmental assessment, (b) civic engagement, (c) psychosocial well-being, and (d) informal care and support was used during Phase 1 of Stage II. The processing of the feedback from Phase 1 resulted in an updated version of the BelRAI Social Supplement (see results), which was used during Phase 2 of Stage II. The items within the instrument are a combination of existing internationally validated items from the interRAI instruments and other not yet validated items that are considered necessary to assess the social context of a person living at home through previous research and in Stage I of this research project [34, 45, 49].

#### BelRAI Screener (BRS)

Although the Social Supplement should be suitable to be used with all interRAI instruments, we chose to link the BelRAI Social Supplement to the BelRAI Screener in its developmental phase. The BelRAI Screener will be fully implemented in Flanders by June 2021 and used by mainly social workers. These social workers are often the first professional initiating home care services for a client. An efficient and wide-ranging first assessment of person’s care needs is therefore crucial [50]. The BelRAI Screener instrument allows for the calculation of a dependency and care complexity index to determine whether a full interRAI assessment is necessary and checks a person’s eligibility to a regional care budget [34].

The BelRAI Screener is a short-form assessment with five questions and respective elaboration modules. It covers ADL, IADL, cognition, psychological problems and behavioral problems in a total of 41 items (See Table 1). As a first step, a professional will evaluate if any, and/or which of the topics the person needs assistance for. When a person is not experiencing issues regarding a certain topic, the items pertaining to that topic will not have to be scored. The BelRAI Screener lets professionals calculate standardized, reliable and validated scores to determine a person’s functional status (measured by the interRAI Activities of Daily Living Hierarchy scale (ADLH) and Instrumental Activities of Daily Living Performance scale (IADLP) [51]), cognitive functioning (interRAI Cognitive Performance Scale 2 (CPS2) [52]), and the presence of behavioral problems (six interRAI items) and psychological problems (five interRAI items) [34]. Table 1 gives an overview of both measures and their content.

**Table 1:** Overview of sections within the BelRAI Screener and BelRAI Social Supplement used in Stage II: Phase 2

BelRAI Social Supplement	BelRAI Screener
**A. Environmental assessment** Number of people living in the household, description of the living arrangements, living conditions, other environmental factors, and access to basic services. **B. Civic engagement** Mobility indoors and outdoors, Use of aids, communication skills, and daytime activities. **C. Psychosocial well-being** Social engagement, unsettled relationships, care denial, feelings of loneliness and social isolation, self-reported mood, strengths, stress, guidance in religion, frequency of social interactions, and financial vulnerability. **D. Informal care and support** Providing care and support to others, Receiving care and support from others, and characteristics of key family caregivers.	**A. IADL***(Performance and capacity)* Meal preparation, ordinary housework, managing finances, managing medications, phone use, stairs, shopping, and transportation. **B. ADL** Personal hygiene, mobility, toilet use, and eating. **C. Cognition** Cognitive skills for daily decision making, short-memory problem, procedural memory problem, and making self understood. **D. Psychological problems** Danger to self, danger to others, inability to care for self, addiction/dependency, and psychiatric symptoms. **E. Behavioral problems** Wandering, verbal abuse, physical abuse, socially inappropriate behavior, inappropriate sexual behavior, and resists care.

### Procedure

#### Stage I: Development of the BelRAI Social Supplement - item generation

Stage I covers the item generation based upon literature, in-depth interviews (*n* = 3) and focus groups with relevant stakeholders (*n* = 9). The collected data were analyzed based upon the directed content analysis approach to define a conceptualization of social context for persons receiving care at home. The different stakeholders in the Flemish care and welfare landscape agreed on a conceptualization of “social context” using 5 main themes: (i) care and support, (ii) physical environment, (iii) life and care goals, (iv) psychosocial well-being, and (v) civic engagement.

A list of practical and content-related criteria was drawn-up to assess which concepts from this multi-faceted framework would be suitable for a Social Supplement to the interRAI instruments. Based on this study, and a continuously updated literature search, items to assess the social context for home-dwelling adults were generated and a first draft of the BelRAI Social Supplement (BSS v1) with a total of 126 items was developed. These results were presented to the project steering committee and their feedback resulted in revised version of the instrument and manual (BSS v2). The second version of the BelRAI Social Supplement was presented to the participants of the interviews and focus groups. They reflected on the selected items, and prioritized relevant items, as well as identified items that were important, but not selected in the first draft. Stage I led to a testable version of the BelRAI Social Supplement (BSS v3) instrument with 155 items (Fig. 1). A detailed paper regarding the methods and findings from Stage I is under review (Van Doren, Hermans and Declercq, unpublished observations).

#### Stage II: Assessment of feasibility and piloting methods

##### Phase 1: preliminary test

In the first phase of Stage II we tested the design of the intervention. The assessors in Phase 1 were social workers active in the same region in Flanders. Each assessor received training on the use of instruments, the software and the ethical procedures. The majority of assessors (*n* = 6) tested the instrument and software with clients of their organization (Family care and Complementary Home Care Services). The other group (*n* = 3) consisted of professional assessors whose job it is to assess – or re-assess - the care needs of individuals in order to check their eligibility for a care benefit and – if needed – contact the appropriate care providers (Social Work Services).

In this study, we include clients of social care services^2^. We included people with chronic diseases (physical or mental) or disabilities, and excluded persons receiving maternity care and/or services for families in a precarious situation, who need help with organizing the household. A person with care needs had to be of legal age (+ 18 years old) and able to give his/her consent to an assessment for this research. Informed consent was obtained from all subjects or, if subjects are under 18, from a parent and/or legal guardian.

The assessors were asked to use the BelRAI Social Supplement for a first practical test during five home visits in the course of 3 months. Afterwards, the participants attended a feedback session to evaluate both the content of the instrument and the manual, and the methods (paper vs. software) used to fill in the instrument. The input given during this feedback session was analyzed to get a clear idea of feasibility, time requirements and to review and refine domains, manual and other training materials. The amended BelRAI Social Supplement (BSS v3) with a total of 101 items was presented to the project steering committee and approved for testing in the entire region of Flanders.

##### Phase 2: large‐scale test

Calls for participation for the second phase of Stage II were sent out to previous participants of Stage I and II of the study (focus group, interviews, follow-up meeting and preliminary test), as well as further disseminated by members of the project steering committee and umbrella home care organizations. A total of 100 care professionals of organizations providing social care services agreed to participate in the study. Each assessor was asked to assess 10 clients with a BelRAI Screener and BelRAI Social Supplement and aim for variability in client profiles. The inclusion and exclusion criteria for the persons being assessed remained the same as in Phase 1.

Six standardized training cycles were organized across Flanders. We explained the aim of the study, the practical requirements and gave an overview of the dates and locations of the different training cycles in an information letter. Participants could use a webpage to register for the location and date of their choosing. A maximum of 25 participants were allowed per training cycle.

Training was provided by a researcher, in collaboration with a specialized BelRAI trainer from the designated training organization in Flanders. And each training cycle consisted of a full day of training and three three-hour discussion groups. These discussion groups combined a teaching and feedback moment and participants were expected to attend at least one of these. The discussion groups were organized at regular intervals after the day of training (approximately 1 month, 3 months and 5 months). This allowed us to continuously follow up on the data collection, to identify problems and present solutions and to get feedback from the professionals. Barriers to further application of the instrument were identified and fine-tuned accordingly. During the last discussion group of each cycle, preliminary data and conclusions were discussed.

Data collection for Stage II (Phase 1 and 2) took place between December 2018 and December 2019. Table 2 provides an overview of the characteristics of the assessors from Phase 2 of Stage II.

**Table 2:** Assessors characteristics during Stage II

Characteristic of the assessors	*n*	*Percentage*
**Gender** (*n* = 100)		
Male	9	9.0
Female	91	91.0
**Organization/Role**		
Family care and Complementary Home Care Services	47	47.0
Social Work Services	52	52.0
Other	1	1.0

#### Stage III: early evaluation of the instrument

##### Data collection

We programmed the BelRAI Screener and BelRAI Social Supplement instruments in Qualtrics Research Core©, a cloud-based survey platform for developing surveys, and collecting data. An anonymous URL-link to the assessments was made available through a password-protected website with accompanying training materials. Professional caregivers also received a hard copy of both the assessments, when the appropriate hardware was not available to them. The assessors were asked to enter the information via pc, laptop, smartphone or tablet using a unique identifier to facilitate data collection. This unique identifier made it possible to connect the data to a specific assessor during data cleaning. Both assessments were completed during a single home visit by a care professional that received the appropriate training. Assessors were encouraged to use their own judgement and to use all sources of information available to complete the BelRAI instruments. Specifically, this means all assessors were told to rely on their own observations and to speak with the person being assessed as well as his or her family members and friends (if available).

##### Data analysis

Data collected in Stage II, Phase 2 were analyzed and evaluated using Excel© and SPSS© for Windows (version 25). Missing values for each item were identified using descriptive statistics. The next step was to shorten the instrument based upon the qualitative and quantitative analyses. An interitem correlation matrix of all the items (from the BelRAI Screener and BelRAI Social Supplement) was computed in order to remove redundant items (*r* > 0.50). Items with very skewed distribution were also reviewed, as these items may not be need-to-know, and could be deleted to shorten the BelRAI Social Supplement.

For the qualitative analyses, we used the assessor’s input during the feedback sessions and discussion groups. Items appraised as nice-to-know rather than need-to-know were considered for removal. When the assessors indicated that they were unable to score an item during the initial home visit, these items were deleted as well.

## Results

A detailed paper regarding the methods and findings from Stage I is under review and titled: Conceptualizing relevant social context indicators for people receiving home care: A multi-method approach in Flanders, Belgium (Van Doren, Hermans and Declercq, unpublished observations). The BelRAI Social Supplement used in the preliminary test was the result of Stage I - Development of the BelRAI Social Supplement.

### Stage II: Assessment of feasibility and piloting methods

#### Phase 1: preliminary test

The data collected during Phase 1 (*n* = 36) was used to identify problems with the practical use and dissemination of the assessment, and the content and structure of the instrument. First, we found that the training of two and a half hours on the content of instrument, the software and ethical procedures was too short to clarify all definitions and procedures. Second, all participants mentioned the extra time necessary after each assessment to input the data from the hard copy into the software. Even though most of the participants had access to a laptop or tablet, they never used the software during their conversation with the person. The participants mentioned that a paper version is easier to use than the research software. The paper enables them to quickly leaf through the instrument during the home visit and skip back and forth to the different sections. The professionals requested an easier-to-use paper version to take with them on home visits.

Professionals also remarked on the length and overall structure of the instrument. Participants indicated that at the start of the data collection the completion of both instruments (BelRAI Social Supplement and BelRAI Screener) took two hours. They suggested to only keep the need-to-know topics in the BelRAI Social Supplement and make the instrument as short as possible. Because they were not familiar with all the topics and questions, they felt “forced” to use the instrument as an interview guideline and gather the information on all topics by just reading the questions aloud. Suggestions of topics to cut from the next version of the BelRAI Social Supplement were approved by the attending participants and project steering committee members. Another topic of concern was the use of certain words and terminology in the instrument and manual. For example, in an item concerning the living conditions of a person the word “filthy” was used. The participants considered such items as stigmatizing and subject to the interpretation of the assessor, and thus not coded consistently.

Linked to length of the instrument, the professionals also mentioned the unnatural flow of the instrument as an issue. The BelRAI Social Supplement encapsulates three different perspectives. Most of the time, the perspective of the assessor is coded. In topics such as ‘self-reported mood’ and ‘feelings of loneliness’ the manual instructs professionals to ask the question verbatim to the person and code their answer. This is in line with other BelRAI instruments, and occurs when a professional is not able to code the item only using their observations. At the end of the section on ‘Informal care and support’, assessors were asked to code the informal caregivers’ answers concerning their view and feelings regarding the current care situation. This was only possible if an informal caregiver was present during the home visit.

Participants were in favor of the different perspectives in one instrument but wanted them grouped in each section. This made it easier to remember which perspectives were applicable to each item. Another suggestion was to use elaboration modules similar to the BelRAI Screener to create a smooth and more logical flow in the instrument. For example, if a person does not provide or receive any informal care and support, then the entire section about informal care and support can be skipped.

These insights resulted in a revised version of the BelRAI Social Supplement with a minimum of 74 and maximum of 101 items, depending on the skip pattern.

#### Phase 2: large‐scale test

A total of 100 professionals assessed 743 adults living at home with both the BRR and the BelRAI Social Supplement. Table 3 provides an overview of client characteristics from Stage II.

**Table 3:** Client characteristics during Stage II

Characteristic of persons assessed with BelRAI Screener & BelRAI Social Supplement	*n*	*Percentage*
**Care dependency** (*n* = 743)		
In need of at least extensive assistance with IADL tasks	539	72.5
In need of at least extensive assistance with ADL tasks	329	44.3
At least moderate cognitive impairment	158	21.3
Depression symptoms present in the last 3 days	431	58.0
Behavioral problems present in the last three days	58	7.7
**Living status** (*n* = 741)		
Non-cohabitation	370	49.8
Cohabitation with adults only	311	42.0
Cohabitation with minors only	20	2.7
Cohabitation with adults and minors	40	5.4
**Type of residence** (*n* = 742)		
House (*single detached, semi-detached or townhouse*)	476	64.2
(Studio) apartment	154	34.2
Other	12	1.6
**Ownership status** (*n* = 741)		
(Co-) owner of the residence (*with or without outstanding loan or mortgage)*	397	53.6
Tenant (*from private individual or public institution*)	291	39.3
Person pays no rent	40	5.4
Other	13	1.8
**Living conditions**		
Home disrepair (*n* = 742)	62	8.3
Squalid conditions (*n* = 743)	34	4.6
Inadequate heating or cooling (*n* = 740)	81	10.9
Lack of personal safety (*n* = 741)	73	9.8
Limited access to home (or rooms in the home) (*n* = 743)	120	16.2
**Use of aids in the last month**		
For mobility (*n* = 742)	472	63.6
For eating (*n* = 741)	103	13.9
For grooming (*n* = 741)	452	61.0
For communication (*n* = 736)	191	26.0
For safety (*n* = 740)	185	25.0
**Feelings of loneliness** (*n* = 736)		
Not lonely	308	41.8
Only in certain situations or triggered by specific events	115	15.6
Occasionally (less than weekly)	117	15.9
Frequently (less than daily)	124	16.8
Daily	72	9.8
**Strengths**		
Having a confidant (*n* = 735)	602	81.9
Consistent positive outlook on life (*n* = 730)	501	68.6
Strong relationship with family (*n* = 734)	558	76.0
Strong relationship with friends (*n* = 734)	393	53.5
**Social interactions in last month**		
Visit to and/or from family members or friends (*n* = 742)	650	87.6
Other interactions *(e.g. telephone or e-mail)* (*n* = 742)	363	85.7
**Financial vulnerability**		
Prevented from receiving essential support because of limited funds (*n* = 740)	134	18.1
**Person is informal caregiver for others** (*n* = 735)		
No	607	82.6
Yes, one other person	78	10.6
Yes, multiple persons	50	6.8
**Receiving informal care from others (*n* = 736)**		
No informal caregivers	71	9.7
One informal caregiver	268	36.4
Two informal caregivers	189	25.7
Three informal caregivers	111	15.1
More than three informal caregivers	97	13.1
**Relation of informal caregiver(s) to person** (*n* = 736)		
Child(-in-law)	507	68.9
Spouse or partner	245	33.3
Parent/Guardian	48	6.5
Other family member	141	19.2
Other (*e.g. friend, neighbor, volunteer*)	105	14.2
**Type of support given to person**		
IADL (*n* = 666)	615	92.3
ADL (*n* = 664)	407	61.3
Childcare (*n* = 659)	74	11.2
Companionship (*n* = 664)	582	87.7
**Caregiver burden**		
Informal caregiver is not able to keep going (*n* = 400)	39	9.8
Informal caregiver is distressed (*n* = 394)	137	34.8

About 73 % of persons assessed with the BelRAI Screener and BelRAI Social Supplement needed at least extensive assistance in instrumental activities of daily living (IADL) such as meal preparation and medication and finances management. Roughly 44.3 % of our sample needed at least extensive assistance in activities of daily living tasks (ADL), such as mobility, eating and personal hygiene. In addition, only 19.9 % showed moderate to severe cognitive impairment, almost 8 % of the sample showed behavioral problems and 58 % had symptoms of depression in the last 3 days

Half of respondents were living alone at the time of the assessment, and minors lived in only 8.1 % of the households. They lived primarily in a house (64.2 %). 34.2 % lived in an apartment or studio. The vast majority of the respondents living in a house (70.2 %) owned the residence (with or without an outstanding loan or mortgage), while 73.2 % of people living in (studio) apartments rented. Almost 70 % of the sample had no issues regarding their living conditions. The most prevalent issue in this sample was a limited access to the home and or rooms in the home (16.2 %). Only 11.6 % scored for more than one issue in their home. The use of physical aids for mobility (63.6 %) and grooming (61.0 %) was most prevalent.

More than a quarter of our sample was on average left alone for 8 h or more in a day, and almost 43 % indicated they - at least occasionally - felt lonely. 81.9 % of the respondents reported to have at least one person to confide in that was not a professional caregiver, and 68.6 % had a consistent positive outlook on life. In the 30 days before the assessment, 87.6 % had received a visit or went to visit family or friends, and about the same percentage of people had other interactions using a telephone or computer. Due to limited financial resources in the past month, 18.1 % of our respondents made trade-offs between necessary aspects for appropriate care and support in their home environment, such as food, shelter, clothing, prescribed medication, sufficient heating or cooling, necessary health care, home care.

The BelRAI Social Supplement’s section on informal care and support is divided in two parts. The first five items are about the person who is being assessed as an informal caregiver to others. 17.4 % of our sample provided care for at least one other person at the time of the assessment. The most common relationships between the person assessed and the person they are caring for were their children(-in-law) (53.9 %) and spouse or partner (34.4 %). The number of persons receiving informal care and support from at least one person was higher (90.3 %), but the most common relationships between the person and informal caregiver were the same for in both parts. 68.9 % of the sample received care from their children(-in-law) and 33.3 % received care from their spouse or partner. The vast majority of informal caregivers in this sample provided companionship (87.7 %) and assistance with IADL-tasks (92.3 %). For a little more than half of the sample, an informal caregiver was present during the assessment (53.0 %). The informal caregivers were asked questions about their ability to keep going and the presence of any feelings of distress. 34.8 % of the assessed informal caregivers admitted to feelings of distress, and almost 10 % indicated they were not able to keep up the level of caregiving.

### Stage III: early evaluation of the instrument

#### Quantitative data-analysis: inter‐item correlation matrix and frequencies

An inter-item correlation matrix indicated that 15 items had moderate to high correlation coefficients within BelRAI Screener and BelRAI Social Supplement (*r* < 0.50). Nine items were part of two internationally validated interRAI scales regarding the living conditions (5 items), and self-reported mood (4 items). Both these scales were considered to be essential for care planning by the participants and were kept in the new version.

The other six items were deleted because of item redundancy. For example; the item B11. Ability to understand others from the BelRAI Social Supplement strongly correlated with a BelRAI Screener item about a person’s ability to making themselves understood (*r* = 0.70). The other items deleted due to moderate to high correlation coefficients were: A13. Access to basic services, B1. Primary mode of mobility, C6. Fearful of family member, C9. Care denial, and C30. Decreased well-being due to limited resources/financial stress.

In the next step of the quantitative approach, the selection of items with very skewed scoring distribution were reviewed. The item A10: Residential instability was a dichotomous item (0 = No, 1 = Yes), and only coded when the person being assessed had no permanent residence in the last 2 years. This could be due to excessive moving, homelessness, etc. Less than 5 % of the sample had issues pertaining to their residential stability. This suggests that the information from this item is not providing ‘new’ or ‘need-to-know’ input for the development of a care plan for almost all clients and can be deleted from the updated BelRAI Social Supplement.

The collection of items regarding a person’s daytime activities (B18 – B27) was based upon the socio-demographics section used in the European Social Survey. These items provided assessors with 10 options to code. These options were as follows: B18. Paid work, B19. Education, B20. Unemployed, actively looking for job, B21. Unemployed, not actively looking for job, B22. Chronically sick or disabled, B23. Retired, B24. Informal caregiver, B25. Volunteering, B26. Other, B27. Refusal. In this sample, all options, except ‘Chronically sick or disabled’ (43.2 %) and ‘Retired’ (69.7 %) were below the 5 % mark. This proved to be a redundant selection of items for the target population of the BelRAI Social Supplement and was deleted as well.

The distribution of the results on the four items concerning a person’s Dutch language proficiency (B12 – B15. Proficiency in listening, speaking, reading, and writing Dutch) and their primary language (B16. Primary language) was also very skewed. The assessors had great difficulty with coding the proficiency items, and almost 95 % of the sample has Dutch as their primary language (Table 4). Several assessors attributed the skewedness of the answers to their own selection bias during the test. They claimed that the length and unfamiliarity of the instrument made it too difficult for them - as well as for the person being assessed - to complete the entire assessment in one conversation when the person had trouble understanding or speaking Dutch. In dialogue with the project steering committee and assessors, we decided to keep these items in the updated BelRAI Social Supplement but create an elaboration section for them to keep a logical flow in the instrument. An item on the presence of problems with the Dutch language is developed to create a gateway to the five items about Dutch language proficiency and primary language.

**Table 4:** Distribution of coding on the items concerning Dutch language proficiency in percentages (Section B of the BelRAI Social Supplement: Civic Engagement)

	Listening	Speaking	Reading	Writing
n	733	736	735	734
Range	0–5	0–5	0–5	0–5
Mean (SD)	4.56 (1.03)	4.47 (1.13)	4.10 (1.56)	3.79 (1.74)
Skewness coefficient	-2.69	-2.33	-1.55	-1.06
A1 Beginner	2	2	7	9
A2 Elementary	2	2	4	6
B1 Intermediate	3	4	7	11
B2 Upper intermediate	6	8	9	8
C1 Advanced	8	7	5	4
C2 Near-native speaker	80	77	70	62

#### Qualitative data‐analysis: feedback from professionals

We found that the design of the training cycles (full day of training and regular discussion groups) was positively evaluated by the professional assessors. The turnout of each of the meetings was good and provided us with a lot of new insights and information on possible issues with the current version of the BelRAI Social Supplement. Several training cycles took place around the same time, but in different provinces. This created the possibility for participants to switch between groups and attend the discussion group that best suited them. Of the 100 trainees, 39 people attended both the full day of training and the following three discussion groups. Almost all assessors (87 %) participated in the training and at least one discussion group. 13 participants were not able to attend any of the subsequent discussion groups due to high turnover or change of position within their organization, long-term sick leave, increased workload and/or conflicting schedules. After each discussion group, the presentations and a summary of the key take-aways of that meeting were added to the website with the training materials.

Five items concerning the level of social engagement and frustration of staff (C1. Person pursues involvement in daily life, C2. Person initiates interactions, C3. Person reacts positively to interactions, and C4. Person adjusts easily to change in routine, C8. Staff frustration) were deleted after discussions, as the assessors indicated that “they are not able to score those items, due their limited time with the person and other professionals’ observations.” These items are validated interRAI items from the Long-Term Care Facility instrument and rely on various moments of interaction with the person being assessed and their caregivers. In the case of the BelRAI Screener and BelRAI Social Supplement, an assessor should be able to score all items using their observations and conversation with the person during one home visit.

Five items were deemed as nice-to-know, but not need-to-know and were removed from the updated version of the BelRAI Social Supplement to create an as-short-as-possible instrument. These nice-to-know items were: A3. Type of residence, B2. Number of days the person went outside, B28. Main daytime activity, C17. Person finds guidance in religion, and D7. Number of care volunteers. The goal of assessors identifying nice-to-know items to make the instrument shorter and leaner, was discussed during the training session. During the subsequent discussion groups these items were reviewed and deliberated. The participants were very vocal about the need to only include items that have a clear and practical use in planning and delivering appropriate and personalized care.

There were very positive reactions to the item B10. Person indicates that they want an (extra) aid. This item has a Yes/No coding scheme, and can be coded when the person indicates they want an (additional) aid to facilitate their mobility, eating, grooming, communication or safety. During the discussion groups several assessors stated that they added this question to their daily assessments as this had proven to shed light on some new information. To enrich on this dummy-variable, an additional item was proposed concerning the different situations in which the person wants to an (extra) aid: mobility, eating, grooming, communication and safety. This item will only be coded when a want is indicated in the previous item, and multiple answer options are possible.

The assessors’ input also shed light on some coding issues, and unclear guidelines. The most recurring questions and remarks were on the timeframe used for coding. For example; the items concerning the use of aids used the coding scheme; 0 = Never, 1 = More than 30 days ago, 2 = 8 to 30 days ago, 3 = 4 to 8 days ago, 4 = In the last 3 days, and 5 = Daily. Assessors indicated that these coding options were too extensive for the topic being assessed. Results also showed that scores 1 to 4 were rarely used. Either the person never used an aid for that situation, or the person used it daily. The assessors requested to shorten the coding scheme to a Yes/No and use a reference period of 30 days.

In summary, 27 items were deleted using a mix of quantitative and qualitive approaches after the large-scale test, and 2 items were added. These insights resulted in an updated version of the BelRAI Social Supplement with a minimum of 46 and a maximum of 76 items, depending on the skip pattern. Section A: Environmental assessment contained 10 items, section B: Civic engagement had a total of 17 items, section C: Psychosocial well-consisted of 22 items, and the section on Informal care and support was 27 items long. In both section B and D, a skip pattern was used to create a smooth and more logical flow.

## Discussion

Developing a Social Supplement to existing interRAI instruments that provides professionals with need-to-know information on a person’s social context is crucial for a shift towards integrated and person-centered health care services. There is general consensus among the stakeholders active in care and welfare settings on the limitations of the biomedical model to determine a person’s care needs [18, 19, 53]. It is clear that this model – with a focus on medical diagnoses and physical functioning alone – lacks crucial data that is necessary for the organizations and the professionals to organize, manage, plan and provide appropriate care [54]. Health and social care providers request standardized and comprehensive tools that document the biological, psychological, environmental and social determinants in a person’s life [34, 44].

Among other things, the WHO’s model of disability (ICF) reasons that the interaction between a person’s psychosocial and environmental determinants and their health condition produces barriers to full participation in society, and in turn influences a person’s experience of their disability [54–59]. The importance lies within the unique impact of social context on persons with disabilities. The exploration of the influence of certain psychosocial and/or environmental determinants on a person’s experiences and daily life and their preferences is something that needs to happen alongside an interRAI assessment.

The aim of this study was to develop the BelRAI Social Supplement as a useful and feasible tool; (i) to raise awareness among the different stakeholders about the importance of the social context indicators, (ii) to gather and provide need-to-know information according to Flemish stakeholders on topics that are known to (in)directly influence a person’s experience of disability [20–23], and (iii) to complement the BelRAI suite of instruments using the same design principles [37].

### In search of balance between rigor and relevance

We used multiple methods to analyze the data as we valued the close collaboration and co-creation with care professionals as of high importance in this study. Additionally, there was clear evidence to suggest selection bias in the quantitative dataset which further warrants this multimethod approach. The imminent implementation of the BelRAI instruments in the home care setting, and the expressed need of home care organizations and professionals for a supplement to assess social context indicators provided us with a healthy breeding ground for successful collaboration and innovation. During the project, there was a continuous search for balance between scientific rigor and practical relevance [60]. We were aware that our research (and the implementation of BelRAI instruments) could only significantly impact the care landscape and its players if we considered the pressing problems in that setting and society at large [61]. In that context, we want to be clear that we deleted items during Stage II and III because they did not fit the scope of our research. However, these items and the themes they are measuring could be relevant and ‘need-to-know’ in other contexts. The criteria used in this study attempted to balance rigor and relevance, while also carefully considering the feasibility, acceptability and viability of the BelRAI Social Supplement instrument using a combination of bottom-up and top-down approach [62].

### Strengths and limitations

 The study design with discussion groups at regular intervals provided us with a large amount of qualitative and quantitative data. Because of the regular meetings and their high attendance rate, issues were quickly solved, and the necessary information was distributed to all assessors using email and our website containing the training materials and reports on each discussion group. Collaboration between a specialized BelRAI trainer and the researchers proved to be a powerful combination to provide each assessor with a standardized, high quality training package and to create a safe environment for the assessors to communicate their problems, views and ideas on the instruments, the training, the software and the preliminary results. High turnover within an organization continues to be an issue effecting the availability and quality of home care services, as well as effectively causing a ‘brain drain’ on specialized assessment instruments [63, 64].

In this study, demographic information (e.g. age, gender, marital status) was not collected due to privacy issues. Also, most of the assessors from Social Work Services did not know the person they assessed prior to the home visit. This implied they had to rely on this one visit to fill out both assessments. In the future, the instrument should be reliable and valid for all adults living at home and will not be restricted to any adult age category. However, the limited demographic information does not give us any confirmation that the sample is representative for all home care clients. Nevertheless, other social context characteristics garnered through the BelRAI Social Supplement show that the sample is quite diverse, with an exception of persons with a low level of Dutch proficiency and (active) families with children present.

The underrepresentation of persons with a low level of Dutch proficiency can be attributed in part to the fact that it is almost impossible (and unethical) to obtain informed consent from a person who is not able to fully comprehend the consent form and/or additional information letter [65, 66]. All stakeholders in our study support the inclusion of the Dutch proficiency items because they consider a person’s language proficiency highly impactful on their access to and utilization of care services as well as their health care experiences. This is in line with previous evidence from scientific studies on the impact of language barriers or illiteracy on participation in health promotion and prevention activities, as well as the level of civic engagement and social integration [67–73].

### Implications for future research

Many of the assessors mentioned they were hesitant to test the BelRAI Social Supplement with their most vulnerable clients as the conversation to complete the instrument frequently took more than an hour and touched on some sensitive topics. Obtaining a better understanding of a persons’ experience during the assessment, could contribute to reducing assessment burden and response bias [74, 75]. In Stage IV: the experience of the person(s) being assessed will be considered during the reliability checks. Research on a person’s and their caregiver’s perception on the assessment process is scarce, but necessary as this can enhance the quality of communication between the professionals and the person(s) they are caring for, as well as help improve training material and intervention strategies [76–78].

## Conclusions

This paper describes the development and testing of a new instrument to supplement existing interRAI instruments: the BelRAI Social Supplement. This instrument aims to gather information on the social context of home-dwelling adults with care needs. The developmental process was done thoroughly, with an exploration of the construct of social context, extensive literature searches, and comprehensive reviews of existing (BelRAI) instruments. All this happened in close collaboration with care professionals and other experts in Flanders, Belgium. Using an iterative process based on the revised MRC-framework, quantitative and qualitative insights helped review and shorten the BelRAI Social Supplement (Fig. 1).

Stage III resulted in a BelRAI Social Supplement with a total of 46 core items and 30 items in elaboration sections, divided over four main sections: (1) environmental assessment; (2) civic engagement; (3) psychosocial well-being, and (4) informal care and support. Stage IV, the final evaluation of the instrument, will help us further examine the BelRAI Social Supplement’s validity. The input from the multiple-methods evaluation will result in a further improved version of the BelRAI Social Supplement, ready for nation-wide implementation.

At the end of the stage IV, a validated and reliable instrument that can assess social context indicators of home-dwelling adults according to the interRAI design principles, will be developed. Our next step will be to translate and further test the structural and cross-cultural validity of the BelRAI Social Supplement in other countries currently using interRAI instruments [79, 80].

## Data Availability

The data that support the findings of this study are available from the Flemish Policy Research Center for Welfare, Public Health and the Family (Flemish Acronym: SWVG) but restrictions apply to the availability of these data, which were used under license for the current study, and so are not publicly available. Data are however available from the authors upon reasonable request and with permission of the Flemish government.

## Abbreviations

ADL: Activities of Daily Living
ADLH: Activities of Daily Living Hierarchy scale
BRS: BelRAI Screener
BSS: BelRAI Social Supplement
CPS2: Cognitive functioning Performance Scale
IADL: Instrumental Activities of Daily Living
IADLP: Instrumental Activities of Daily Living Performance scale
ICF: International Classification of Functioning, Disability and Health
MRC: Medical Research Council
WHO: World Health Organization

## Footnotes

1 Belgium is a federal state, composed of communities and regions. The Communities have powers for culture, education, the use of languages and matters relating to the individual which concern on the one hand health policy (curative and preventive medicine) and on the other hand assistance to individuals (protection of youth, social welfare, aid to families, immigrant assistance services, etc.). For more information on Belgian’s state structure, see: https://www.belgium.be/en/about_belgium/government/federale_staat/structure.

2 Clients of social care services in Flanders are mainly (about 50 %) persons over 80. Other types are people who are chronically ill or have disabilities.
